# A comprehensive assessment of oral health, swallowing difficulty, and nutritional status in older nursing home residents

**DOI:** 10.1038/s41598-023-47336-w

**Published:** 2023-11-14

**Authors:** Kuei-Ru Chou, Mao-Suan Huang, Wan-Chun Chiu, Yi-Hsiu Chen, Yu-Yoh Chen, Qian Xiao, Suh-Ching Yang

**Affiliations:** 1 School of Nursing, College of Nursing, Taipei Medical University, Taipei, Taiwan; 2 Center for Nursing and Healthcare Research in Clinical Practice Application, Wan Fang Hospital, Taipei Medical University, Taipei, Taiwan; 3 Department of Nursing, Taipei Medical University-Shuang Ho Hospital, New Taipei City, Taiwan; 4 Psychiatric Research Center, Taipei Medical University Hospital, Taipei, Taiwan; 5 Neuroscience Research Center, Taipei Medical University, Taipei, Taiwan; 6 School of Oral Hygiene, College of Oral Medicine, Taipei Medical University, Taipei, Taiwan; 7 School of Nutrition and Health Sciences, College of Nutrition, Taipei Medical University, Taipei, Taiwan; 8 Research Center of Geriatric Nutrition, College of Nutrition, Taipei Medical University, 250 Wu-Hsing Street, Taipei, 11031 Taiwan; 9 Department of Nutrition, Wan Fang Hospital, Taipei Medical University, Taipei, Taiwan; 10 Graduate Institute of Health and Biotechnology Law, Taipei Medical University, Taipei, Taiwan; 11 Nutrition Research Center, Taipei Medical University Hospital, Taipei, Taiwan; 12 School of Gerontology and Long-Term Care, College of Nursing, Taipei Medical University, Taipei, Taiwan

**Keywords:** Health care, Medical research, Risk factors

## Abstract

Declines in oral consumption and swallowing function are common reasons which may elevate the risk of malnutrition in the older adults. This study aimed to provide valuable information and contribute to the existing body of knowledge in this field as well as highlight the importance of a comprehensive assessment of oral health, swallowing function, and nutritional status in long-term care residents. This was a cross-sectional study. Thirty-nine participants were recruited from a nursing home. The comprehensive assessment was evaluated in participants, including oral health (Oral Health Assessment Tool (OHAT)), swallowing function (Functional Oral Intake Scale (FOIS) and Eating Assessment Tool (EAT)-10), and nutritional status (Mini Nutritional Assessment-Short Form (MNA-SF). The average age of participants was 80.4 ± 11.7 years, and 46% of these older adults were found to be at the risk of malnutrition. There was a negative correlation between the OHAT and MNA-SF scores. In addition, subjects with poor oral health (OHAT score = 5~8), oral consumption of a modified diet (FOIS score = 4~6), and reduced swallowing function (EAT-10 score ≥ 3) were more likely to be at risk of malnutrition. A comprehensive evaluation of oral health and swallowing function was closely connected with the nutritional status of older nursing home dwellers.

## Introduction

Malnutrition can occur at any age but is especially prevalent in people older than 60 years of age^1–4^. In recent years, the malnutrition prevalence has been increasing worldwide due to the aging of the population and increasing prevalence of age-related pathological conditions^5^. A recent meta-analysis that included over 110,000 older persons underscored the malnutrition rate possibly ranging 6~29.4% (95% confidence interval (CI), 21.7–36.9) based on the healthcare setting^6^. Many cases of malnutrition go undiagnosed, leading to increased morbidity and mortality, prolonged hospital stays etc.^7,8^. Early recognition and treatment of malnutrition are not only beneficial for older subjects’ health but also can reduce overall costs to healthcare systems^9^. Involuntary weight loss is one of the risk factors of malnutrition in older adults^10^. Robbins popularized a mnemonic device consisting of nine Ds, which included dentition and dysphagia, to describe common causes of weight loss in geriatric populations^11^. In this scenario, dysphagia, and oral health are closely linked by sharing common pathophysiological pathways, disabling sequelae, and malnutrition.

The Mini Nutritional Assessment (MNA) is a clinical assessment tool for grading the nutritional status and evaluating malnutrition risks in patients or individuals that do not need a dietitian. The MNA is well-validated with high sensitivity, specificity, and reliability based on anthropometric measurements, the general health status, a dietary questionnaire, and a subjective assessment of health and nutrition^12^. Moreover, previous studies suggested that the MNA should be included in geriatric assessments, and they proposed a minimum dataset for nutritional interventions^7,8,12^. Although the MNA can be completed within 10~15 min, it is not frequently used in acute-care settings due to its lengthy completion time^12–15^. To further reduce this short time burden, a six-question MNA short-form (MNA-SF) was developed by identifying a subset of questions from the full MNA^15^.

To assess oral health, the Oral Health Assessment Tool (OHAT) is a useful tool for informal caregivers without specific training to indicate whether the person they care for should visit an oral healthcare professional^16^. Furuya et al. indicated that poor oral health was associated with a poor nutritional status and nutrition intake methods^17^. Hugo et al. also demonstrated that a poor nutritional status was associated with worse oral health in aged care residents^18^. Regarding the assessment of swallowing ability, the Eating Assessment Tool (EAT-10) and Functional Oral Intake Scale (FOIS) are common methods for non-professionals^19,20^. The EAT-10 is a self-administered questionnaire for dysphagia screening, with each item scored from 0 to 4. Wakabayasi and Matsushima indicated that dysphagia assessed by the EAT-10 was associated with the nutritional status assessed by the MNA in older individuals requiring long-term care^21^. Moreover, the FOIS was originally validated to determine changes in the occurrence and severity of dysphagia in an adult population over time^22^. Simon et al. emphasized that malnutrition screening could identify head and neck cancer patients who were at risk of malnutrition and subsequently needed to be referred to a dietician for nutritional support^23^. However, a relatively small number of studies has assessed the relationships among oral health, swallowing ability, and the nutritional status. The main reason may be that no comprehensive assessment tools have been established for long-term care residents or community-dwelling older adults.

Our hypothesis was that independent-living older people with poor oral health as assessed by the OHAT or at high risk of dysphagia as evaluated by the EAT-10 and FOIS would be more likely to be at risk of malnutrition based on an assessment by the MNA-SF. Therefore, the aims of this study were to highlight the importance of a comprehensive assessment of oral health, swallowing function, and nutritional status in long-term care residents. Additionally, this study also tried to raise awareness about the nutritional status of institutionalized older adults and identify factors, such as oral health and swallowing function, that may influence the nutritional status.

## Results

### Characteristics of study participants

**Figure 1:**
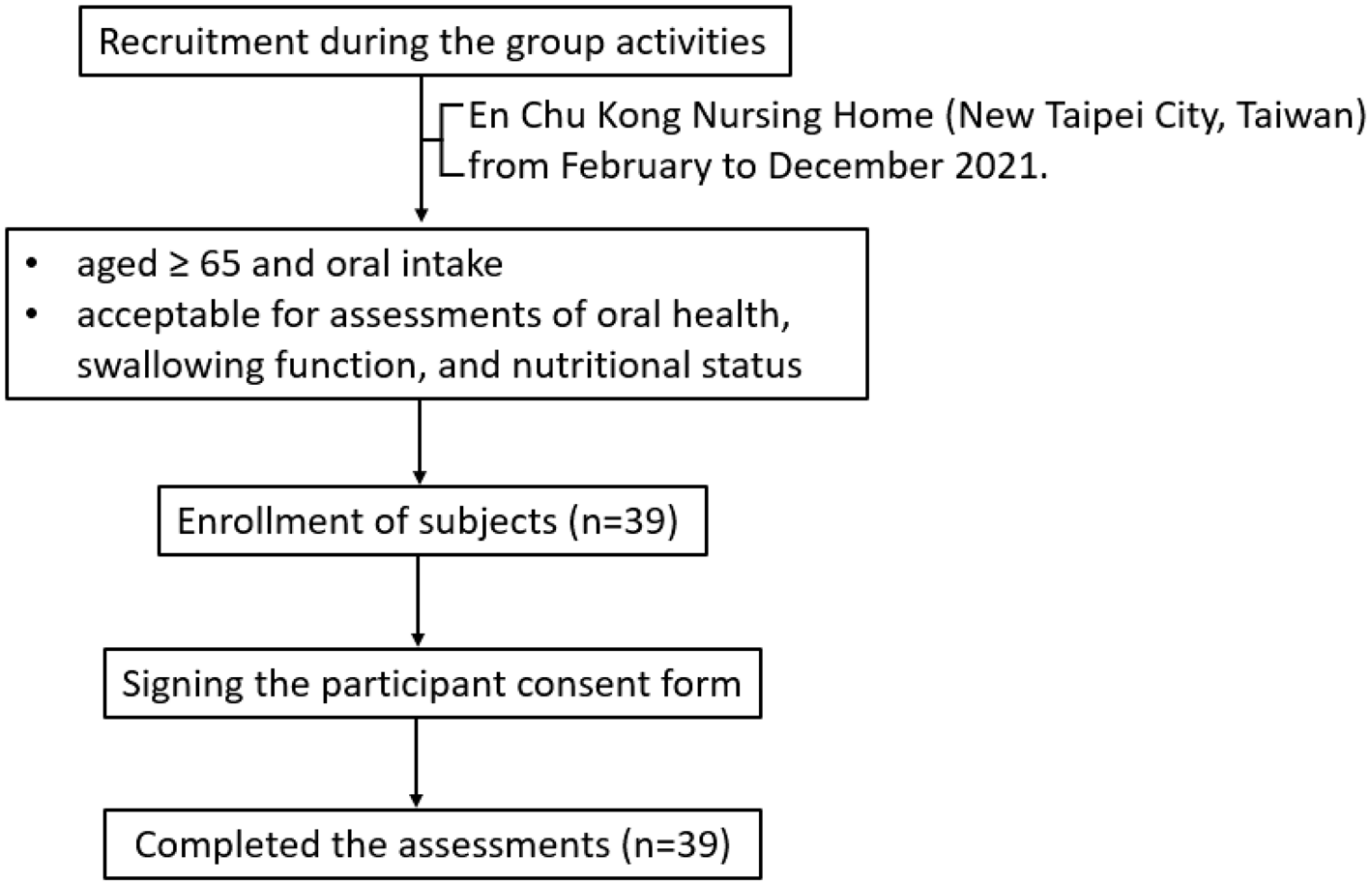
Study flow diagram for the assessments of oral health, swallowing function, and nutritional status in older nursing home residents.

**Figure 2:**
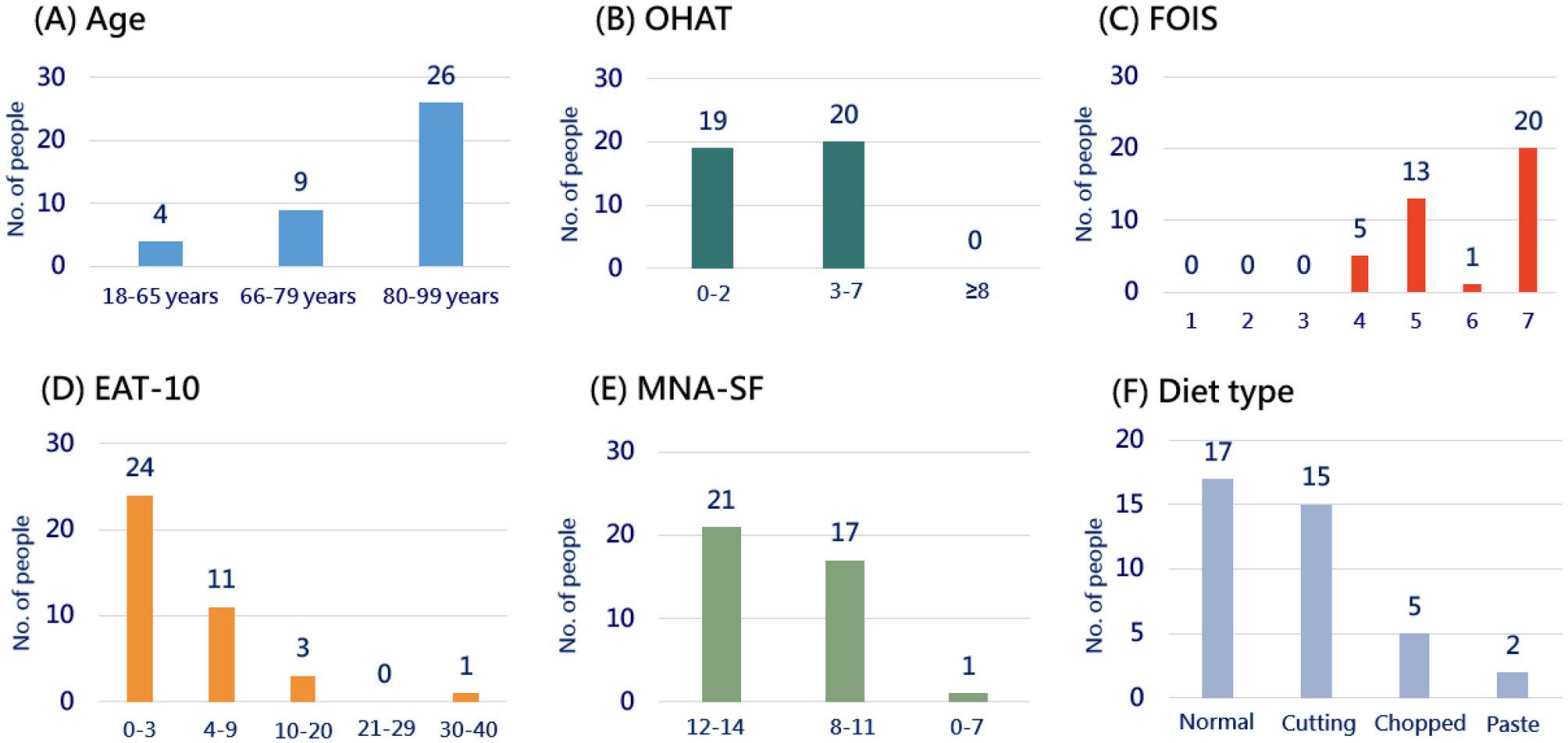
The number of people for each screening item. (**A**) The age distribution of participants; (**B**) OHAT, Oral Health Assessment Tool; (**C**) FOIS, Functional Oral Intake Scale; (**D**) EAT-10, Eating Assessment Tool; (**E**) MNA-SF, Mini Nutritional Assessment–Short Form; and (**F**) diet type provided to participants.

Thirty-nine participants entered the study (Fig. 1), and the characteristics of participants are represented in Tables 1 and 2. In addition, the number of people in each screening items is shown as Fig. 2. The average age of the participants was 80.4 ± 11.7 years, and the average BMI was in the normal range (20~24 kg/m^2^) as defined by the Health Promotion Administration, Ministry of Health and Welfare, Taiwan (Table 1). Although participants' fasting blood sugar levels were high, they did not meet the criteria for diabetes (Table 2). Five subjects had high fasting blood glucose levels of > 126 mg/dL, which is defined as diabetes. All participants took food by mouth and were provided with the texture-modified diet, including normal, cut, chopped, or paste (Fig. 2C, F). Approximately 49% of subjects had an OHAT score of < 2 (Fig. 2B). Around 38% of subjects had an EAT-10 score of > 3 (Fig. 2D). In addition, 46% of participants had an MNA-SF score below 12 indicating a risk of malnutrition (Fig. 2E).

**Table 1:** Characteristics of study samples with respect to MNA-SF scores. Data are expressed as the mean ± standard deviation. The Shapiro–Wilk's test was used to test the normality; data were compared by the Mann–Whitney U-test or *t*-test. A Chi-squared test or fisher’s exact test were performed for categorical variables. OHAT, Oral Health Assessment Tool; FOIS, Functional Oral Intake Scale; EAT-10, Eating Assessment Tool; MNA-SF, Mini Nutritional Assessment–Short Form.

Variable	All (*n* = 39)	Well-nourished (MNA-SF = 12~14, *n* = 21)	Risk of malnutrition (MNA-SF = 0~11, *n* = 18)	*p* value
Anthropometric measurements				
Age (years)	80. 4 ± 11.7	81.19 ± 9.99	79.39 ± 13.59	1
Gender				
Male (*n*, %)	17, 43.6%	10, 47.6	7, 38.9	0.584
Female (*n*, %)	22, 56.4%	11, 52.4	11, 61.1	
Height (cm)	156.98 ± 10.64	156.34 ± 10.63	157.72 ± 10.91	0.6920
Weight (kg)	58.57 ± 11.30	61.12 ± 10.23	55.59 ± 12.04	0.1294
Body-mass index (kg/m^2^)	23.70 ± 3.63	24.97 ± 3.10	22.21 ± 3.71	0.0060
Diet texture type				
Normal (*n*, %)	17, 43.6%	11, 52.4	6, 33.3	0.1290
Cut (*n*, %)	15, 38.5%	9, 42.9	6, 33.3	
Chopped (*n*, %)	5, 12.8%	1, 4.8	4, 22.2	
Paste (*n*, %)	2, 5.1%	0, 0.0	2, 11.1	
Oral hygiene and health				
OHAT (score)	2.51 ± 2.04	2.19 ± 2.23	2.89 ± 1.78	0.2296
OHAT score 3–7 *(n,%)*	20, 51.3%	8, 38.1%	12, 66.7%	0.1110
Swallowing function				
FOIS (score)	5.92 ± 1.18	6.19 ± 1.08	5.61 ± 1.24	0.1213
FOIS score below 7 *(n,%)*	19, 48.7%	8, 38.1%	11, 61.1%	0.2049
EAT-10 (score)	4.62 ± 6.83	2.81 ± 4.76	6.72 ± 8.30	0.0635
EAT-10 score more than 2 *(n,%)*	18, 46.2%	7, 33.7%	11, 61.1%	0.1130
Nutritional assessment				
MNA-SF (score)	11.59 ± 1.94	13.05 ± 0.80	9.89 ± 1.41	< 0.0001

**Table 2:** Biochemical parameters with respect to MNA-SF scores. Data are expressed as the mean ± standard deviation. The Shapiro–Wilk's test was used to test the normality, and data were compared by the Mann–Whitney U-test or t-test. A Chi-squared test was performed for categorical variables. MNA-SF, Mini Nutritional Assessment–Short Form; ALT, alanine aminotransferase; ALT, aspartate aminotransferase; BUN, blood urea nitrogen.

Variable	All (*n* = 39)	Well-nourished (MNA-SF = 12~14, *n* = 21)	Risk of malnutrition (MNA-SF = 0~11, *n* = 18)	*p* value
Albumin (g/dL)	3.86 ± 0.40	3.88 ± 0.30	3.84 ± 0.50	0.6497
AST (U/L)	22.41 ± 8.22	25.43 ± 9.27	18.89 ± 5.04	0.0025
ALT (U/L)	17.04 ± 11.26	20.67 ± 10.22	12.8 ± 11.19	0.0017
BUN (mg/dL)	20.79 ± 12.60	22.19 ± 14.84	19.17 ± 9.51	0.4047
Creatinine (mg/dL)	1.26 ± 0.91	1.40 ± 1.06	1.10 ± 0.70	0.0492
Total cholesterol (mg//dL)	153.90 ± 31.38	144.43 ± 23.76	164.94 ± 36.01	0.0401
Triglyceride (mg/dL)	119.77 ± 66.25	122.00 ± 82.29	117.17 ± 42.79	0.5923
Fasting blood glucose (mg/dL)	103.15 ± 52.70	102.19 ± 41.33	104.28 ± 64.77	0.6019
Na (mEq/L)	140.23 ± 3.77	140.48 ± 4.04	139.94 ± 3.51	0.5593
K (mmol/L)	3.98 ± 0.44	3.93 ± 0.39	4.05 ± 0.49	0.4131

When participants were divided into two groups based on the MNA-SF score, there were no differences in age, gender, height, or weight between well-nourished participants (MNA-SF 12~14) and participants at risk of malnutrition (MNA-SF = 0~11). Well-nourished participants had higher BMIs compared to participants at risk of malnutrition (Table 1). There were no significant differences in either oral health or swallowing function test, but well-nourished participants had a trend of lower OHAT and EAT-10 scores (Table 1).

### Biochemical parameters

There were no differences in albumin, BUN, TGs, fasting glucose, plasma Na, or plasma K levels between the two groups. Well-nourished participants had higher AST, ALT activities and creatinine level, but they were still in a normal range. The TC level was significantly lower in the well-nourished group compared to the group at risk of malnutrition (Table 2).

### Correlation between different parameters

Table 3 illustrates correlations between the different parameters. Compared to the OHAT, there was a negative correlation in the scores of the FOIS and MNA-SF, and a positive correlation of the EAT-10 score and diet type. In addition, the FOIS was negatively correlated with the EAT-10 and diet type. The EAT-10 showed a positive correlation with diet type. The score of the MNA-SF was positively associated with weight, BMI, AST, and ALT activities. The blood Na level was only positively correlated with the FOIS score.

**Table 3:** The correlations between related variables and screening items. Data were analyzed by the Shapiro–Wilk's test and Spearman correlation. BMI, body-mass index; OHAT, Oral Health Assessment Tool; FOIS, Functional Oral Intake Scale; EAT-10, Eating Assessment Tool; MNA-SF, Mini Nutritional Assessment–Short Form; ALT, alanine aminotransferase; ALT, aspartate aminotransferase; BUN, blood urea nitrogen.

Variable	OHAT		FOIS		EAT-10		MNA-SF	
	*r*	*p* value	*r*	*p* value	*r*	*p* value	*r*	*p* value
Age	0.0536	0.7458	− 0.1769	0.2815	0.0234	0.8876	0.1164	0.4804
Height	− 0.0120	0.9422	0.0357	0.8294	− 0.0389	0.8139	− 0.1113	0.5001
Weight	− 0.2312	0.1568	0.0740	0.6542	− 0.2294	0.1601	0.3169	0.0493
BMI	− 0.2493	0.1259	0.0576	0.7278	− 0.2797	0.0846	0.6104	< 0.0001
OHAT	–	–	− 0.4807	0.0020	0.3622	0.0235	− 0.3280	0.0415
FOIS	− 0.4807	0.0020	–	–	− 0.6731	< 0.0001	0.2707	0.0955
EAT-10	0.3622	0.0235	− 0.6731	< 0.0001	-	-	− 0.3130	0.0523
MNA-SF	− 0.3280	0.0415	0.2707	0.0955	− 0.3130	0.0523	-	-
Diet type	0.4565	0.0035	− 0.7846	< 0.0001	0.6574	< 0.0001	− 0.3106	0.0543
Albumin	− 0.2032	0.2146	0.1390	0.3986	0.0095	0.9541	− 0.0467	0.7777
AST	− 0.0919	0.5779	0.2149	0.1888	− 0.2675	0.0996	0.5298	0.0005
ALT	− 0.1804	0.2716	0.2359	0.1483	− 0.1613	0.3267	0.5038	0.0011
BUN	0.1338	0.4168	0.0150	0.9280	− 0.0067	0.9678	0.1816	0.2685
Creatinine	− 0.0015	0.9925	0.1020	0.5367	− 0.1004	0.5430	0.3106	0.0543
Total cholesterol	− 0.0408	0.8051	0.0783	0.6359	− 0.1955	0.2329	− 0.2839	0.0799
Triglyceride	− 0.2509	0.1235	− 0.0583	0.7247	− 0.1377	0.4033	− 0.0567	0.7317
Fasting blood glucose	0.0873	0.5972	− 0.1750	0.2867	− 0.1126	0.4948	− 0.0322	0.8458
Na	− 0.2560	0.1157	0.3542	0.0269	− 0.1052	0.5241	0.1854	0.2586
K	0.1360	0.4155	0.1385	0.4069	− 0.0360	0.8301	-0.2186	0.1873

### ORs related to risk of malnutrition or malnutrition

Variables independently associated with the screening MNA and adjusted by means of the multivariate logistical regression are listed in Table 4. Older people with a higher OHAT score were more likely to be at risk of malnutrition according to screening by the MNS-SF (power value = 0.373). In addition, subjects with a lower FOIS score were more likely to have malnutrition compared to subjects who had no swallowing problems (FOIS = 7) (power value = 0.276). Participants scoring 3 or more on the EAT-10 were more likely to be at risk of malnutrition compared to those who scored 0~2 (power value = 0.378). Moreover, after adjusting for age, gender, and BMI, a similar trend was observed (Table 4). Although the odds ratios suggest a higher risk of malnutrition with higher OHAT score, lower FOIS score, and higher EAT-10 score, the small sample size results in a lower power value. Therefore, further confirmation is needed in the future regarding this aspect.

**Table 4:** Crude and adjusted odd ratios (ORs) of variables related to a risk of malnutrition based on the MNA-SF scores. The adjust ORs were adjusted for age, gender, and the body-mass index. OHAT, Oral Health Assessment Tool; FOIS, Functional Oral Intake Scale; EAT-10, Eating Assessment Tool; MNA-SF, Mini Nutritional Assessment–Short Form.

Screen items		Crude	Adjusted
	Score	OR (95% CI)	OR (95% CI)
OHAT	0~2	1	1
	3~7	3.250 (0.87, 12.137)	2.349 (0.553, 9.977)
FOIS	7	1	1
	4~6	2.554 (0.700, 9.311)	4.179 (0.874, 19.989)
EAT-10	0~2	1	1
	3 and more	3.143 (0.846, 11.671)	2.75 (0.606, 12.472)

## Discussion

The present results indicated that 46% of independent older adults in a long-term care facility were at the risk of malnutrition. As an initial screening tool, the MNA-SF is especially useful for the older adults who can provide accurate and reliable information about themselves^2^. If malnutrition is recognized early, a nutritional intervention can be effectively implemented^7,9^. Thus, regular monitoring of weight loss and screening for signs of malnutrition should be conducted for older individuals who are not malnourished or who are at risk of malnutrition to prevent overall incidences of malnutrition^26^.

In this study, OHAT scores were negatively correlated with MNA-SF scores even the r value was low (Table 3). In addition, persons with a higher OHAT score were more likely to be at risk of malnutrition (Table 4). This result indicated that poor oral health might be the one of possible reasons for inducing malnutrition in the older subjects. Poor oral health and poor oral function have been implicated as risk factors for a poor diet^27–29^. A systematic review demonstrated that functional teeth units and the mean number of teeth present were significantly associated with the nutritional status^30^. There are eight categories in the OHAT, including natural teeth and dentures, and it takes slightly less time to complete than the Brief Oral Health Status Examination (BOHSE) studies^31^. Therefore, the OHAT was evaluated to be a quick, reliable, and valid screening assessment tool for use in long-term care facilities.

All participants were orally consuming food in this study (Fig. 2, Table 1). Although there was no association between the FOIS and MNA-SF or between the EAT-10 and MNA-SF (Table 3), those who were consuming a texture-modified diet and who had poor swallowing function were more likely to be at risk of malnutrition (Tables 3, 4). It was indicated that dysphagia by the EAT-10 is associated with the nutritional status of older individuals requiring long-term care^21^. Dysphagia is the inability to swallow and is related to low nutritional intake^32,33^. Older adults are more likely to suffer from dysphagia, since ageing reduces the swallowing function at the oral, pharyngeal, and esophageal stages^34–38^. Modifying the consistency of solid foods or liquids is one of the mainstays of compensatory interventions for patients with dysphagia^39^. The goal of diet modification is to improve the safety of oral consumption and thus maintain safe and adequate oral intake of foods or liquids^33^. It is possible, however, that older people with dysphagia may have inadequate nutrition because of low acceptability and poor adherence to modified foods and liquids^40^. It was indicated that 91% of nursing home residents placed on modified diets were placed on overly restrictive diets^40^. As a result, older people living in nursing homes need to be provided with appropriately modified diets based on an accurate diagnosis of their swallowing ability to prevent malnutrition. On the other hand, the 3-Ounce Water Swallow Challenge Screening Protocol and the Toronto Bedside Swallowing Screening Test (TOR-BSST) are also valid tools for evaluating swallowing function; however, they must be administered by well-trained professionals^41,42^.

The present study showed that all blood parameters were in the normal range, including well-nourished participants who had significantly higher AST and ALT activities, and creatine levels and lower TC levels compared to participants at risk of malnutrition (Tables 2, 3). The reasons why elderly who was at the risk of malnutrition showed the significant higher blood TC level might be the permanent, untreated, intrinsically higher concentration of cholesterol in the individual subject^43^. In addition, it has been reported a J- or U-shaped relationship between total cholesterol level and mortality in the older and general populations as well as in various patient populations^44^. Due to the high prevalence of malnutrition and chronic diseases among the older adults, it is crucial to pay close attention to the levels of total cholesterol in their blood. On the other hand, the blood albumin level, which is commonly used an indicator of malnutrition, was unchanged whether malnutrition was present or not (Table 2). The small sample size might be the main reason why a difference could not be observed; however, other malnutrition-related indicators such as prealbumin should be measured^45^. Moreover, the biochemical data were gathered during a routine health physical examination, thus it is recommended to perform more blood analyses in the future, such as high-density lipoprotein cholesterol (HDL-C) or low-density lipoprotein cholesterol (LDL-C).

The World Health Organization (WHO) emphasized the importance of an interprofessional framework in a super-ageing society^46^. Furthermore, Japanese have the highest life expectancy in the world, and they proposed that an interprofessional competency framework, education, and collaboration were necessary for the healthcare of older adults^47^. On the other hand, across studies, approximately 20% of nursing home residents had some form of malnutrition. However, malnutrition definitions were variable, and prevalence ranged 1.5~66.5%^48^. The main reason is the lack of professional manpower and negligence in performing overall health assessments. To truly tackle the issue of malnutrition in nursing home settings, a consistent and routine set of screening tools for eating function and nutritional status in residents is strongly recommended. After professional interpretation, the screening tools used in this study can be completed by caregivers, then a simple, routine screening model can be established.

To our knowledge, this is the first report to complete comprehensive assessment of oral health, swallowing function, and nutritional status, and to investigate associations among poor dentition, dysphagia, and malnutrition. Based on this study, a comprehensive assessment system must be established to prevent malnutrition in older residents in nursing homes and long-term care facilities. Despite its strengths, the present study also has several limitations. First, this study design was a cross-sectional model with a small sample size, which limited the data interpretation such as the low r value between OHAT and MNASF. Multiple influences, outliers and randomness are also the possible reasons for the low r value. Accordingly, longitudinal, or interventional studies with a more-extensive sample size are required to determine causal relationships among oral health, swallowing function, and the nutritional status in nursing home-dwelling older adults. Second, blood biochemical variables such as the distribution of blood cells, HbA1C, and anemia-related indicators were not included in the analysis. Finally, several screening tools are still used in related research, such as the Geriatric Oral Health Assessment Index (GOHAI), Dysphagia Risk Assessment for the Community-Dwelling Elderly (DRACE), Malnutrition Universal Screening Tool (MUST), etc.^49–51^. Although we emphasize that the tools used in this experiment had a characteristic of simple operation, it is necessary to use different tools to explore correlations between eating functions and the nutritional status of older adults.

In conclusion, the present results indicated that around 46% of older adults living in this nursing home were at the risk of malnutrition based on the MNA-SF evaluation. According to the assessment of oral health (OHAT) and swallowing function (FOIS and EAT-10), oral consumption of a modified diet and swallowing problems and the nutritional status seemed to be related that must be confirmed in the future. These findings suggest that routine evaluation is necessary as a strategy of nutritional management for preventing older adults’ malnutrition. This study also conveys the idea of bringing attention to the nutritional status of institutionalized older adults, identifying influencing factors, and emphasizing the importance of evaluating these factors including oral health and swallowing functions.

## Methods

### Study design

This was a cross-sectional study which was approved by the Taipei Medical University (TMU)-Joint Institutional Review Board (protocol ID: N202107030; ClinicalTrials.gov ID: NCT05013918). Participants were recruited from the En Chu Kong Nursing Home (New Taipei City, Taiwan) in February to December 2021. Regarding the recruitment of participants, the institutional nursing staff explained the details of the experiment during group activities to recruit voluntary participation from older adults. The inclusion criteria for recruitment were individuals aged ≥ 65 who were capable of oral intake and willing to undergo assessments of oral health, swallowing function, and nutritional status. All procedures were conducted according to principles expressed in the *Declaration of Helsinki*. Written informed consent was obtained by the participant's own willingness.

### Training

Data collection included anthropometric measurements, oral health and swallowing function evaluation, and nutritional status assessment. All assessments were conducted by nursing home nurses and caregivers who were well-trained and certified by a dentist, speech therapist, and dietitians. The nurses and caregivers underwent a 2-day training program, including lectures and practicum. After the training program, nurses and caregivers had to complete the comprehensive assessment for older nursing home residents and be approved by experts. On the other hand, the biochemical parameter data were collected from reports of regular health examinations of nursing home residents.

### Anthropometric measurements

Height and body weight were collected from a regular health examination. Then, the body-mass index (BMI) was calculated by the formula: weight (kg)/height (m)^2^. The food texture was recorded by the caregiver through daily care. For the data analysis, normal food texture was defined as 0, cutting was defined as 1, chopping was defined as 2, and paste texture was defined as 3.

### Oral health

Oral health was assessed by the OHAT. A score of 0 = healthy, 1 = oral changes, or 2 = unhealthy was given in each of the assessment categories, and scores of the eight categories were summed to give a total score^24^. The assessment categories include lip, tongue, gum and tissues, saliva, natural teeth, dentures, oral cleanliness, and dental pain^25^. A higher total score indicated poor oral health.

### Swallowing function

Swallowing function was determined by a subjective assessment tool, the EAT-10. The assessment contained 10 questions, and each question was scored 0~4. A score of 0 represented no functional abnormality, while a score of 4 represented severe functional abnormalities. The summed EAT-10 total score ranged 0~40, with a score ≥ 3 indicative of dysphagia^22^.

The FOIS was used to assess the function of oral intake objectivity which included seven items. Levels 1 through 3 relate to varying degrees of non-oral feeding, and levels 4 through 7 relate to varying degrees of oral feeding which considered diet modifications^23^.

### Nutritional assessment

The nutritional status was examined by the MNA-SF. The MNA-SF contains anthropometric measurements (BMI and weight loss), mobility, a dietary questionnaire, neuropsychological problems, and acute diseases. A score of 0~7 indicated malnutrition, a score of 8~11 indicated as a risk of malnutrition, and a score of 12~14 indicated a normal nutritional status^25^.

### Biochemical parameters

Biochemical parameter data were collected by a regular health examination in the nursing home. The collected data included a nutritional status-related indicator (albumin), liver function index (aspartate aminotransferase (AST) and alanine aminotransferase (ALT)), renal function index (blood urea nitrogen (BUN) and creatinine), lipid profile (total cholesterol (TC), triglycerides (TGs)), glucose, and minerals (Na and K).

### Statistical analysis

Statistical analyses were performed with GraphPad Prism Software vers. 5.0 (GraphPad Software, La Jolla, CA, USA) or SPSS vers. 22 software (SPSS, Chicago, IL, USA), and all values are expressed as the mean ± standard deviation (SD) or percentage for categorical variables. The normality of the population was tested by the Shapiro–Wilk's test. Differences among groups were compared by the Mann–Whitney U-test (nonparametric) or *t*-test (parametric). Correlations of the MNA-SF, OHAT, FOIS, and EAT-10 with each other were analyzed by Spearman correlations. Chi-squared test and odds ratios (ORs) were analyzed by SPSS software. The Chi-squared test or fisher’s exact test was performed for categorical variables. Crude and adjusted ORs were represented with 95% confidence intervals (CIs) and calculated for the MNA-SF, oral health, and swallowing function. The value for analyzing the adjusted OR included age, gender, and BMI. A *p* value of < 0.05 was considered statistically significant. The independent variable was well-nourished and risk of nutrition (MNA-SF) in the crude odds ratio, while age, gender and BMI were added in the adjusted odds ratio. G*Power software vers. 3.1 (Heinrich-Heine-Universität Düsseldorf, Düsseldorf, Germany) was used to calculate the power value of odds ratios.

### Ethics approval

This study was approved by the Taipei Medical University (TMU)-Joint Institutional Review Board (N202107030) and registered in ClinicalTrials.gov (NCT05013918).

## Data Availability

The datasets generated during and/or analyzed during the current study are available from the corresponding author on reasonable request.
